# Wrist Arthrodesis Using a Trapezoidal Wedge Graft from the Distal Radius and a Low-Profile Reconstruction Plate

**DOI:** 10.1007/s43465-023-00884-9

**Published:** 2023-04-17

**Authors:** Anil K. Bhat, G. Mithun Pai, Ashwath M. Acharya, Amruth Manohar

**Affiliations:** Department of Hand Surgery, Kasturba Medical College, Manipal, Manipal Academy of Higher Education, Manipal, 576104 India

**Keywords:** Wrist, Fusion, Arthrodesis, Brachial plexus, Rheumatoid arthritis

## Abstract

**Background:**

Various intramedullary or dorsally based fusions have been utilised to perform wrist arthrodesis. Although the dorsal plate is rigid and well constructed, the standard of care was replenishing the arthrodesis site with an iliac crest bone graft. Due to the high morbidity of the donor site, alternatives such as distal radius bone grafts have gained popularity. In this study, wrist arthrodesis was performed with a locally accessible trapezoidal wedge graft from the distal radius and a low-profile reconstruction plate to evaluate the radiological and functional outcome.

**Methods:**

We retrospectively reviewed 22 wrists, 14 brachial plexus injuries, 4 post-traumatic injuries, and 4 rheumatoid arthritis patients, with a mean follow-up of 31 months. Union was evaluated on radiography. The functional outcomes were evaluated using a visual analog scale incorporated into a questionnaire.

**Results:**

All 22 fusions united successfully, with a mean duration of 12 weeks and a wrist position of an average 17.5 degrees of extension and 6 degrees of ulnar deviation. The aesthetics of the wrist showed the most significant change, and overall satisfaction levels increased.

**Conclusions:**

A locally accessible cortico-cancellous graft from the dorsum of the radius is a reliable alternative to an iliac crest or carpal bone graft with high potential for the union. It also serves as a stable strut in our construct, allowing us to use a low-profile reconstruction plate. The Reconstruction (3.5 System) plate can be used safely with excellent results and a low implant prominence or breakage risk.

## Introduction

Different techniques and implants, such as rush rods, Steinman pins, K wires, staples, and cancellous screws, have been used to perform wrist fusion through intramedullary or dorsal approaches for various indications, including arthritis and paralytic wrist [1–4]. A dorsal plate was recommended subsequently because it enabled rigid fixation, minimised postoperative immobilisation, facilitated rapid fusion, and reduced complications such as nonunion and hardware failure [5]. Extensor tendinitis around the terminal portion of the plate is one of the documented potential consequences, which would demand surgical removal of the implant [6]. A precisely engineered, contoured, low-profile plate may reduce the likelihood of tendonitis [7]. As a result, the AO/ASIF compression pre-contoured, the terminally tapered plates were developed, with irritation of tendons and synovial inflammation developing less frequently around the plate than with conventional dynamic compression plates [8]. However, the wrist arthrodesis plate is expensive and carries a risk of fatigue fracture [8]. Even if the dorsal plate is rigid and the implants are better constructed, the standard of care is still to use cancellous bone from the iliac crest to fill the fusion site [8]. Due to the high morbidity of the donor site [9], which includes long-lasting pain, hematoma formation, infection, and nerve damage, alternatives such as fusion without bone graft [10], proximal row carpal graft [11], and distal radius bone graft [12] have become more popular. Only individuals with low bone quality remain candidates for iliac crest grafts [13]. A pain-free wrist is a goal for an arthritic wrist, whereas strengthening wrist stability allows for coordinated grasping and releasing of objects in a paralytic wrist, such as a brachial plexus injury [14]. In this study, wrist arthrodesis was performed using a locally accessible trapezoidal wedge of the cortico-cancellous graft from the dorsal aspect of the distal radius and a low-profile reconstruction plate. Even though various configuration of distal radius graft has been used in the past, the combination of a low-profile reconstruction plate and a trapezoidal graft to achieve wrist fusion has not been reported. The purpose of the study was to assess the radiological and functional outcome of such a procedure.

### Methodology

Institutional ethical board clearance was obtained before evaluation. Between 2016 and 2020, 22 patients at our tertiary care centre underwent wrist arthrodesis using the technique of a dorsal low-profile reconstruction plate augmented with a trapezoidal wedge of cortico-cancellous bone graft from the distal end of the radius by a dorsal approach. This retrospective study included 19 male and 3 female patients, ranging in age from 24 to 72 years, who had been followed for at least a year. The right wrist was involved in 17 cases, while the left was involved in only 5. In 18 patients, the dominant wrist was involved. Four cases of post-traumatic, 4 cases of rheumatoid arthritis, and 14 cases of brachial plexus injuries were indications for arthrodesis of the wrists in our patients.

In patients with global brachial plexus palsy, joint instability and a lack of donors for reanimation were the indications for arthrodesis to improve residual function in the hand and digits. In contrast, pain was the primary indication for wrist arthrodesis in individuals with rheumatoid arthritis and post-traumatic arthritis. Rheumatoid arthritis patients with good bone density were chosen for this wrist arthrodesis method. Patients’ occupational profiles included manual labourers, clerical professionals, retired workers, and homemakers. Patients with poor bone quality and individuals requiring alternate bone grafts were excluded from the study. A questionnaire based on Van Heest and Strothman’s research [15] evaluating wrist fusion in cerebral palsy assessed aesthetics, functionality, and contentment with wrist arthrodesis. At the final follow-up (minimum 1-year follow-up), all 22 patients were personally examined and interviewed with the help of a VAS (visual analog scale) incorporated into this validated questionnaire (Table 1).

**Table 1:** Questionnaire for postoperative functional assessment following wrist arthrodesis

Sl.no	Domains of the questionnaire
1	Did the procedure make your wrist look better? (Aesthetics)
2	Did the procedure make your wrist more functional? (Functionality)
3	Has the operation made your day-to-day tasks simpler? (Everyday care)
4	Did the wrist fusion procedure make you more hygienic? (Cleanliness)
5	How has your function changed due to your wrist fusion? (Change in function)
6	Are you still experiencing wrist pain? (Pain)
7	How satisfied are you with the results of your wrist fusion surgery? (Satisfaction)

### Technique

**Fig. 1:**
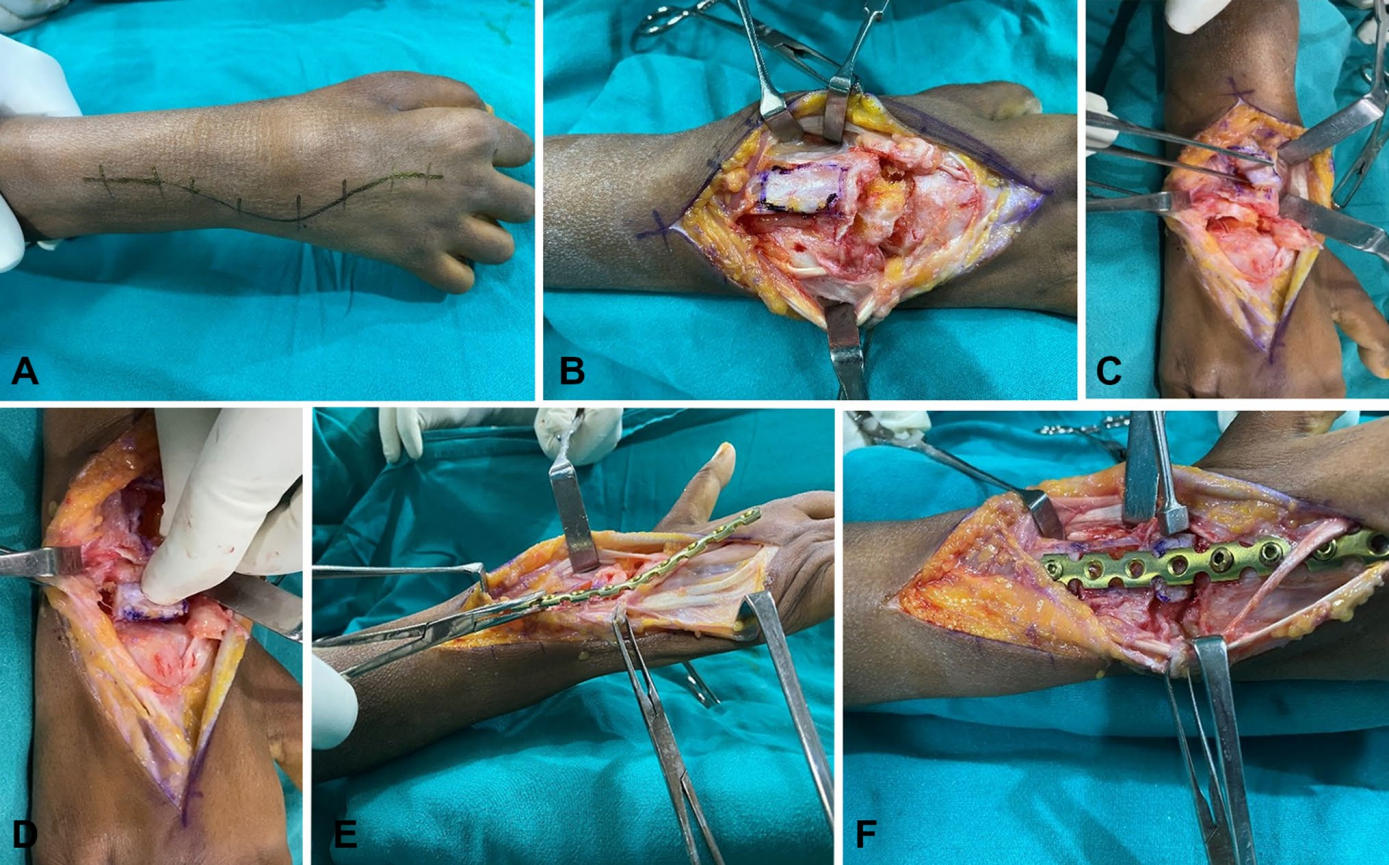
Our technique of harvesting the strut cortico-cancellous graft from the dorsum of the wrist and fusion of the wrist using a low-profile 3.5 recon plate. **A** A curvilinear incision, **B** marking for graft harvest, **C**, **D** the bone graft is used to horizontally span the radiocarpal region on the trough formed by the radiocarpal area, **E**, **F** the low-profile plate moulded as per the requirement

A standard dorsal approach was used to expose the wrist. An 8-cm-long, curvilinear incision was made from the lower end of the radius to the distal third of the metacarpal (Fig. 1A). The wrist joint was exposed through the interval between the third and fourth extensor compartments by incising the extensor retinaculum above the fourth dorsal compartment and finally by longitudinally exposing the wrist capsule. The insertion of extensor carpi radialis brevis was subperiosteally elevated, and the interosseous fascia over the third metacarpal was incised. The lower end of the radius and carpals, including the scaphoid, lunate, capitate, and third metacarpal base, were exposed subperiosteally. A significant degree of decortication was performed to remove the cartilage surface of the radiocarpal joint and the carpal bones. A small portion of the posterior interosseous nerve was excised just proximal to the articular surface of the radius in very painful wrists. During the procedure, the third carpometacarpal joint was not fused. However, the dorsal portion of the joint was freshened and replenished with cancellous grafts later to add up to solid fusion.

A trapezoidal cortico-cancellous graft of an average size of 3 × 4 cm was harvested from the dorsum of the lower end of the radius, 1 cm proximal to the radius articular surface (Fig. 1B, C). The bone graft is used to horizontally span the radiocarpal region on the trough formed at the radiocarpal area after removing the cartilage of the carpal bone and dorsiflexing the wrist to about 15^0^ (Fig. 1D). The graft is interposed between the radius and the carpal bones as a press fit, reducing the possibility of any graft displacement. This also eliminates the need for additional locking screws to be inserted into the graft or carpal bone to establish a stable construct, as used in a wrist fusion plate. Fixation is performed with a 3.5 mm reconstruction plate and a 10 or 12-hole plate that can be shaped with a plate bender.

**Fig. 2:**
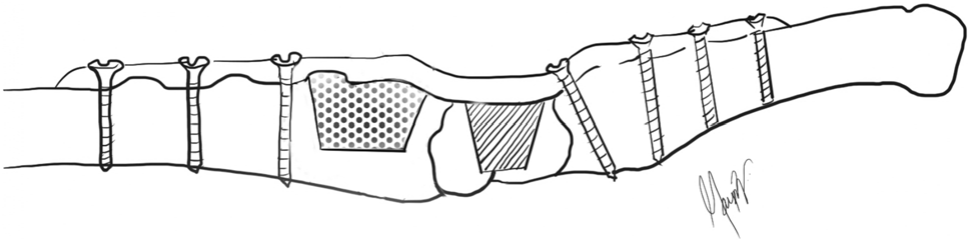
Diagrammatic representation of our wrist arthrodesis technique

Typically, plate length is calculated to obtain a minimum of six cortices of bone in the third metacarpal and six to eight cortices in the lower end of the radius. The wrist is fixed at 10 to 15 degrees dorsiflexion and 5-degree ulnar deviation (Fig. 1E, F). After positioning the plate, the metacarpal’s most distal holes were drilled first. This is secured with the cortical screw to fine-tune the position and compression of the plate to the bone. The plate was subsequently fixed to the radius shaft (Fig. 2). The drill hole through the metacarpal portion of the plate must be in the sagittal plane. If not, the radius fixation will cause rotational deformity of the third metacarpal. Accurate plate alignment in the dorsal midline of the metacarpal is essential to avoid malrotation and maximise grip strength potential. The capsule and extensor retinaculum was repaired with interrupted sutures, exteriorising the tendons to avoid direct contact with the plate. The skin was sutured together with absorbable sutures.

### Rehabilitation and Assessment

After the surgery, a well-padded below-elbow cast was applied for 6 weeks. We immediately began active and passive motions to promote motion in the fingers and elbow. A volar, below-elbow thermoplastic wrist splint was provided at 6 weeks, and mobilisation began. The protective splint was removed when X-rays revealed union (trabeculae spanning the fusion site), commonly observed at an average of 12 weeks following the treatment. A radiographic assessment at 6, 12, and 16 weeks was done to determine union at the arthrodesis site. Patients were followed up for a minimum of 1 year later.

## Results

**Fig. 3:**
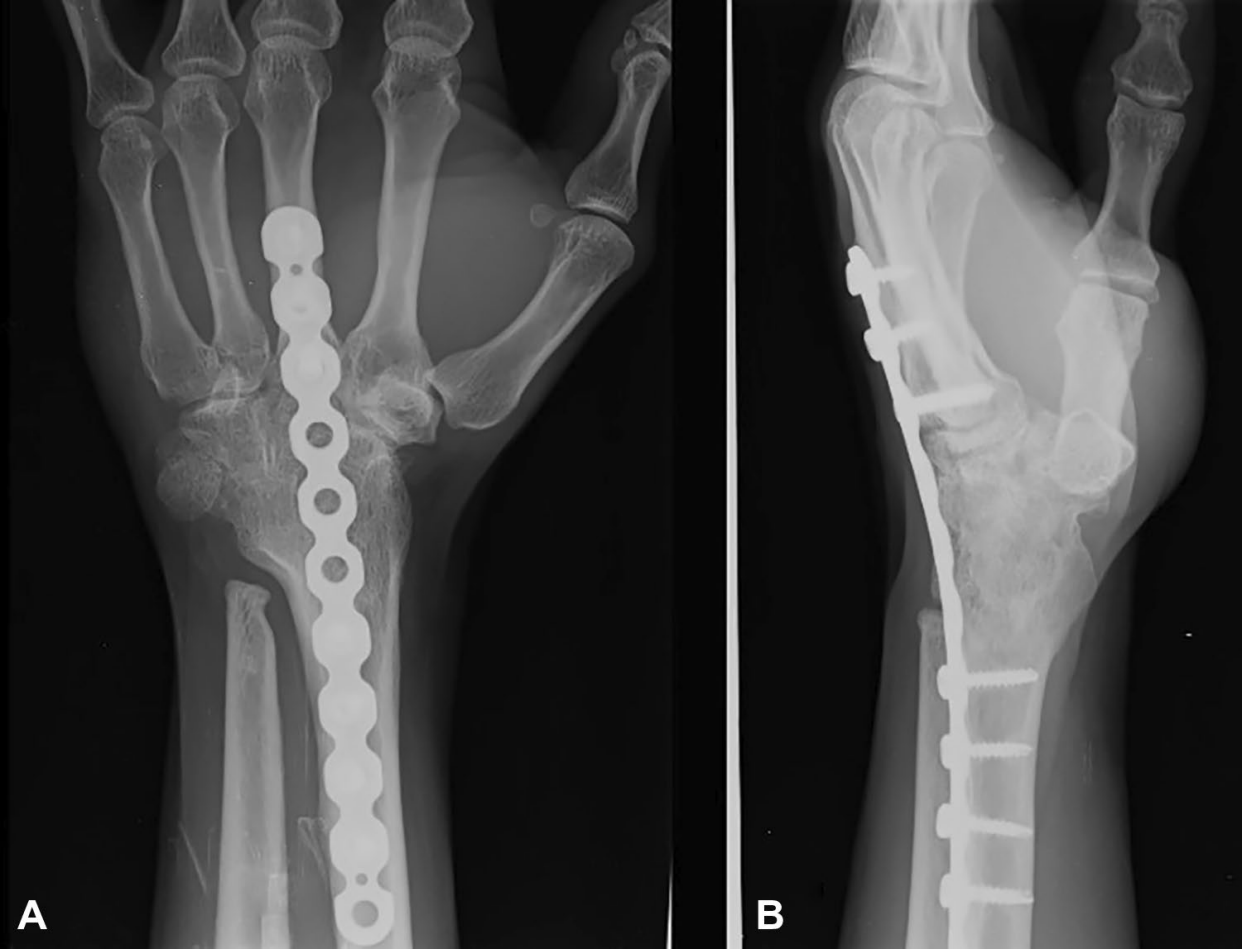
Two years following wrist fusion in a patient with rheumatoid arthritis shows union on radiograph with maintained position and filling up of the donor site

The average duration of follow-up was 31 months, ranging from 12 to 48 months. Follow-up X-rays showed that all 22 wrists had fused completely at an average time of 12 weeks (range 12 to 16 weeks). There was no incidence of extensor tendonitis in the initial follow-up (16 months) due to the low-profile nature of the implant. At the most recent follow-up, ranging from 12 to 48 months, none of the X-rays showed any signs of plate loosening, hardware prominence, or screw backouts. In five patients, Darrach’s procedure was performed at the time of arthrodesis, of which two cases were rheumatoid arthritis, and three were post-traumatic cases. An FCU (flexor carpi ulnaris) sling was used to stabilise the ulna stump in these patients. Patients’ average postoperative hand position following wrist arthrodesis was 17.5 degrees of extension (with a range of 14.5 degrees to 20 degrees of extension), with 6 degrees of ulnar deviation (range 5 degrees to 8 degrees) (Fig. 3). The donor site healed well in all the cases without any morbidity.

The subjective questionnaire was graded using a VAS (visual analog scale) scoring system ranging from Zero to 10. Improvement was indicated by a score higher than five, the relative decline was indicated by a score below five, and a score of five was considered neutral. 80% of patients indicated that their appearance had improved. 68% reported feeling an improvement in the ease of their day-to-day care. Regarding their cleanliness, 42% of patients reported an improvement. Seventy percent of the patients experienced a reduction in pain. Sixty-two percent of patients said they were content with the operation and delighted. Forty percent of patients indicated that their wrist function had improved, while 60% were ambivalent about the condition. In contrast, when patients were asked how their function had altered after surgery, none reported that it was worse than before surgery, and 70% of them said that it was at least somewhat improved (Table 2).

**Table 2:** Comparing average VAS scoring for various domains of a questionnaire for different indications

Domains of questionnaire	Brachial plexus injuries (mean)	Rheumatoid arthritis (mean)	Post-traumatic arthritis (mean)
Aesthetics	6.8	8.6	8.3
Functionality	5.2	7.3	7.6
Everyday care	5.2	6.8	7.6
Cleanliness	5.8	8.6	8.3
Change in function	5.2	7.4	7.2
Pain	6.2	7.3	9
Satisfaction	5.2	8.2	8.6

## Discussion

Intramedullary stabilisation augmented with staples or Kirschner wires [3] and internal fixation with plates through a dorsal approach is described as techniques for wrist fusion [5]. These techniques are reliable for treating advanced pan-carpal degenerative pathologies. The common indications for wrist fusion are patients with severe rheumatoid arthritis, trauma-related osteoarthritis, spasticity, brachial plexus palsy, post-infection articular arthritis, and failed implant arthroplasty [4].

The dynamic compression plate is commonly utilised to fuse the wrist because it facilitates stable fixation and compression with excellent stability. Since its introduction, dynamic compression plating has become the most prevalent technique for reconstructing injured wrists caused by various disorders [5]. Extensor tendon synovitis, which causes pain around the end of the plate, is one of the most noticeable sequelae [6]. Although AO plates have been routinely utilised for wrist arthrodesis since their introduction by Heim and Pfeifer in 1974, 10% of patients have problems that require implant removal [8]. Therefore, a tapered AO wrist arthrodesis plate with a 3.5 mm proximal screw, a 2.7 mm distal screw, and a particularly tapered end for the metacarpal shaft was devised [16].

**Fig. 4:**
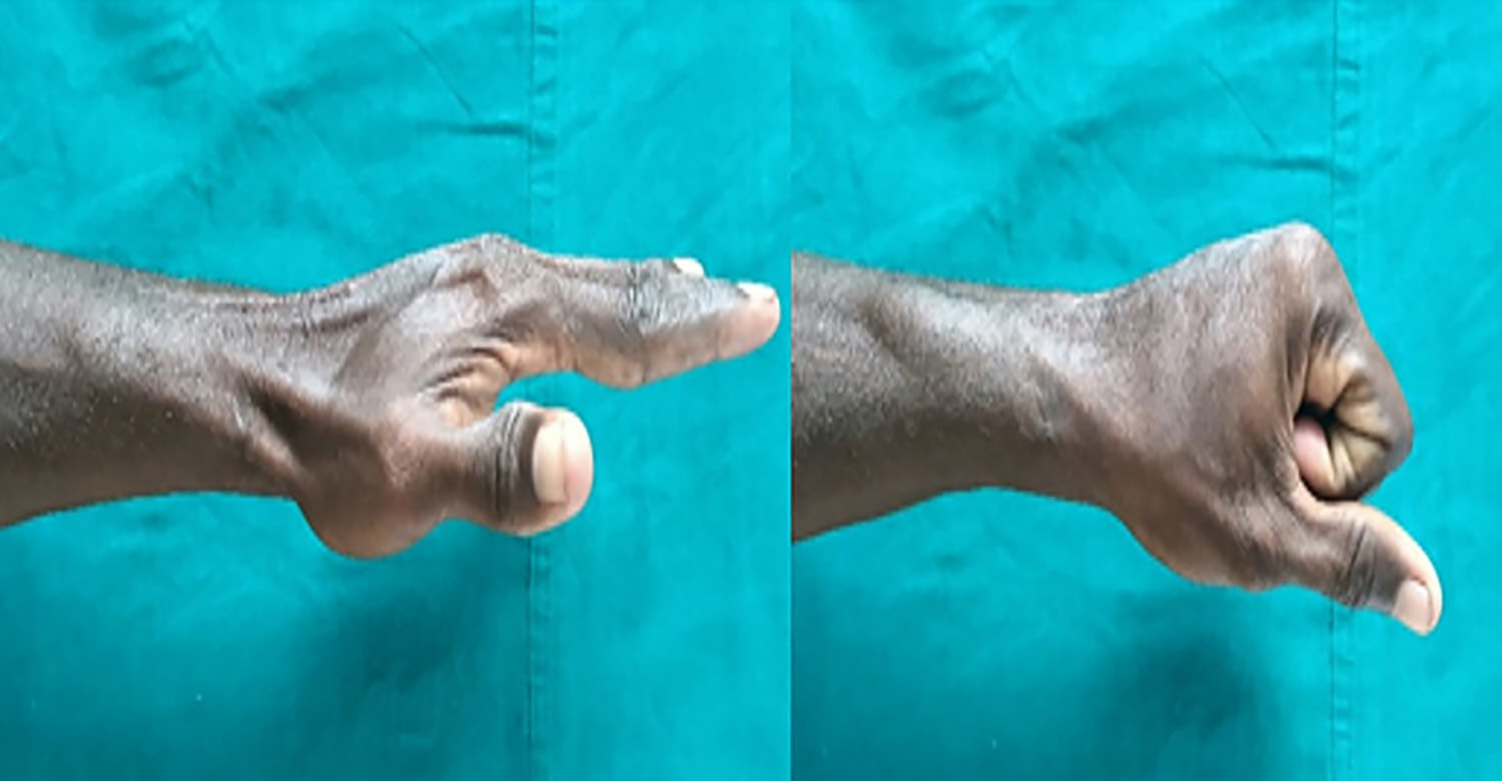
Three years following wrist arthrodesis in a patient with rheumatoid arthritis with no evidence of implant prominence

In the most extensive series of wrist fusions performed with an AO wrist arthrodesis plate, 42 patients with post-traumatic arthritis and 3 with rheumatoid arthritis were included. The results were excellent regarding union and patient satisfaction in six cases. Additional analysis of 17 fusion procedures employing an AO wrist arthrodesis implant, including 2 patients with rheumatoid arthritis, revealed successful bone healing and excellent patient satisfaction[5]. In two cases, however, plate-related problems occurred, including extensor tendinitis and a screw hole fracture. Since it was difficult to shape the implant precisely enough to achieve a modest wrist extension and ulnar deviation, the pre-contoured feature was devised. Our study demonstrated that the low-profile 3.5 mm reconstruction plate, which can be moulded using a plate bender, achieved an efficient wrist fusion. The dorsal approach provided ease in harvesting a trapezoidal-shaped graft from the distal radius that interposed well between the radius and the carpal bones. The plate has a low elastic modulus, thereby providing stability, has a hexagonal construction that enables the formation of vascular bridges across the fusion site with enhanced healing, has tolerance to multi-directional mechanical forces across the fusion site, oval holes for compression mode, and locking screws that achieve compression. The reconstruction plate is thinner and less prominent than the conventional AO wrist arthrodesis plate. Our study reported no plate-related complications, including implant breakage. There was 100% fusion noted in our study (Fig. 4).

Due to the donor site morbidity associated with iliac crest graft harvest, which includes persistent discomfort, hematoma formation, infection, and nerve damage, alternative methods have gained popularity [6]. Among these operations are fusion without a bone graft [10], bone graft harvesting from the lower end of the radius [12], proximal carpal row graft [5], and ulna head graft [17]. Bruce Gill was the first to use the distal radius as a graft in 1923. Stein (1958) later referred to this procedure as the “Gill turnabout radial graft” [18]. Evan [19] fashioned the entire distal end of the radius into a wedge and inserted it into an open-ended carpus. Therefore, this arthrodesis shortens the forearm to permit a good union. However, in two instances, wedge displacement was seen [19]. Clayton (1965) combined the intramedullary Steinman pin fixation with radial graft [20]. In 1987 [21], Wood refined the method by placing the harvested distal radius graft into the third metacarpal base and stabilising it using tension band wire. Sorial [22] trimmed the cortico-cancellous graft from the distal end of the radius into a strut to move distally into a trough in the carpus and third metacarpal base, then stabilised it with a dynamic compression plate [22]. Weiss [23] executed wrist fusion using a pre-contoured dynamic compression plate and a radius bone graft. Extensor tendinitis, characterised by inflammation and pain across the extensor tendons, was detected at the distal end of the implant in four individuals, necessitating the removal of the plate [23]. In 1998, Chang performed wrist arthrodesis with a procedure involving a radius bone graft and minimal internal fixation, achieving a high fusion rate [12] The dorsal radius graft was rotated 180 degrees in the coronal plane by Luboshitz [24], and cancellous or cortical screws were used to secure each end [24]. In the studies mentioned above, the success rate of total wrist fusion using a rigid plate and a bone graft from a local source was excellent regarding the union, as seen from the table (Table 3) Ours is a unique technique and the first of its kind. It utilises the distal radial trapezoidal graft as a press fit and stabilises with a low-profile reconstruction plate, achieving 100% union.

**Table 3:** Comparison of the studies utilising radius bone graft for wrist arthrodesis

Studies utilising distal radius graft	Sample size	Implant used	Distal radius graft	Fusion rate(%)	Time to fusion
Evan et al. [19]	18	No implant	Wedge lower end radius	89	–
Wood et al. [21]	16	K wire with tension band principle	As a bone plate	100	10 weeks
Sorial et al. [22]	18	AO dynamic compression plate	Corticocancellous strut	100	12 weeks
Weiss et al. [23]	28	Dynamic compression plate	Corticocancellous slices	100	10 weeks
Chang et al. [12]	8	K wire with tension band principle	Corticocancellous strut	100	–
Luboshitz et al. [24]	25	Cortical/cancellous screws	180^0^ rotation of radius graft	84	14 weeks
Our study	22	3.5 Reconstruction plates	Trapezoidal wedge of cortico-cancellous graft	100	12 weeks

**Fig. 5:**
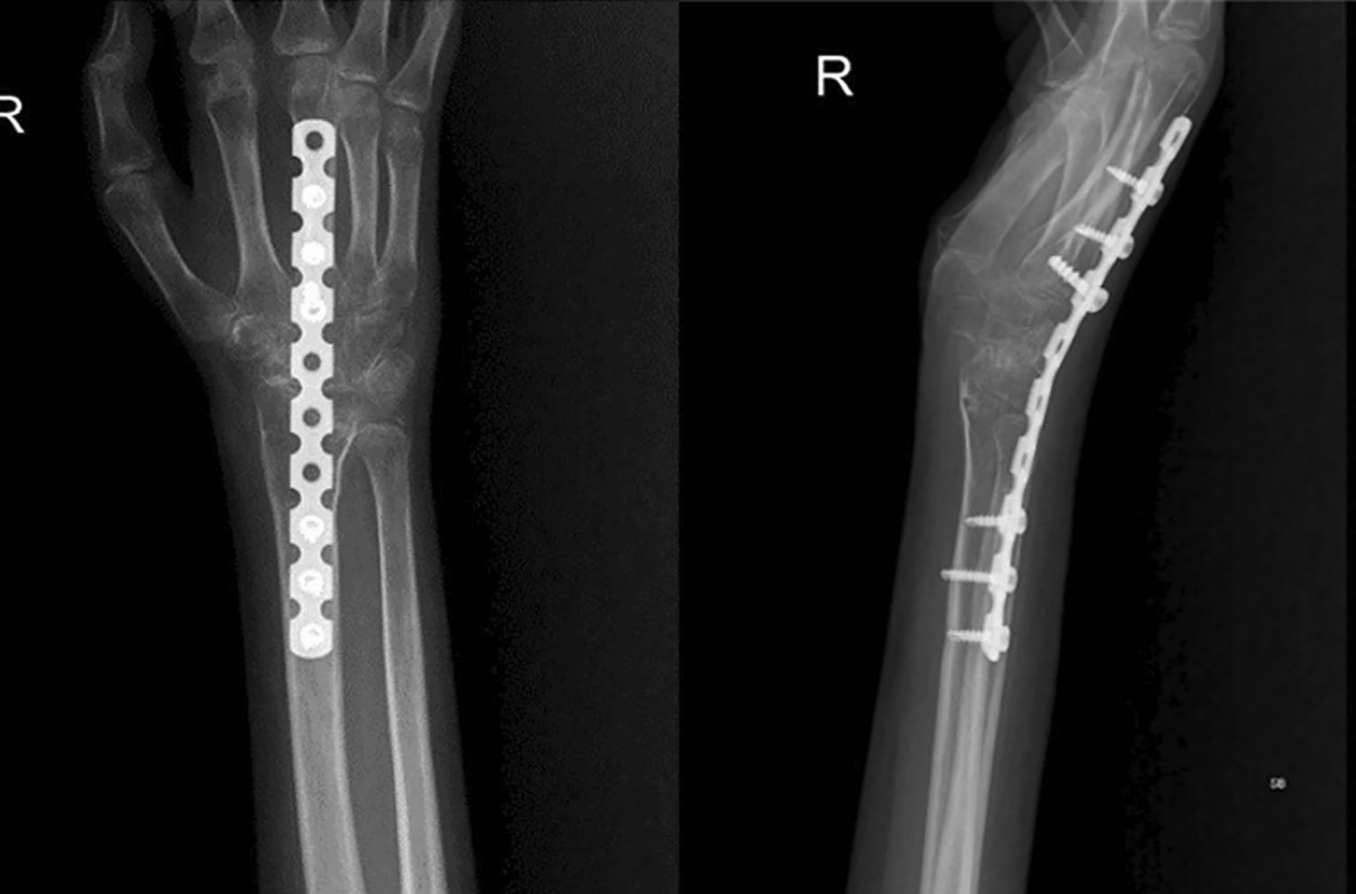
Three years following wrist fusion in a patient with brachial plexus palsy shows union on radiograph with the maintained position with filling up of the donor site

Patients with substantial osseous defects or low bone quality should still be considered for iliac crest bone grafting to augment arthrodesis. We used the trapezoidal strut of cortico-cancellous graft from the dorsum of the lower end of the radius, which is press fit horizontally into the trough created by the carpal bone decortication and the extension provided by the low-profile reconstruction plate. We did not encounter extensor tendonitis, plate prominence, implant breakage, or subsequent implant removal. By the final follow-up, the donor site had completely healed in all cases (Fig. 5).

Terzis et al. [25] used an AO dynamic compression plate and iliac crest bone graft to fuse the wrists in 61 patients with global brachial plexus injury. They achieved a pain-free, stable wrist with improved upper extremity function. Addosooki et al. [26] performed wrist fusion using a dynamic wrist arthrodesis plate and cancellous graft from Lister's tubercle. The fusion worked effectively with a double-functioning free muscle transfer, enabling patients with global brachial plexus palsy to achieve a considerable hand grip. They also concluded that fusing the wrist to the second metacarpal enhanced overall hand function and the range of motion in the fingers. Giuffre et al. [27] reported that patients with global brachial plexus palsy could benefit from wrist fusion (using a dorsal locking wrist fusion plate) combined with other reconstructive surgeries to improve the function of the hand. In our series, 14 patients with brachial plexus injuries underwent wrist fusion, and the union was observed in all the patients with better functioning of transferred free muscle as described in Table 2 (Fig. 5).

**Fig. 6:**
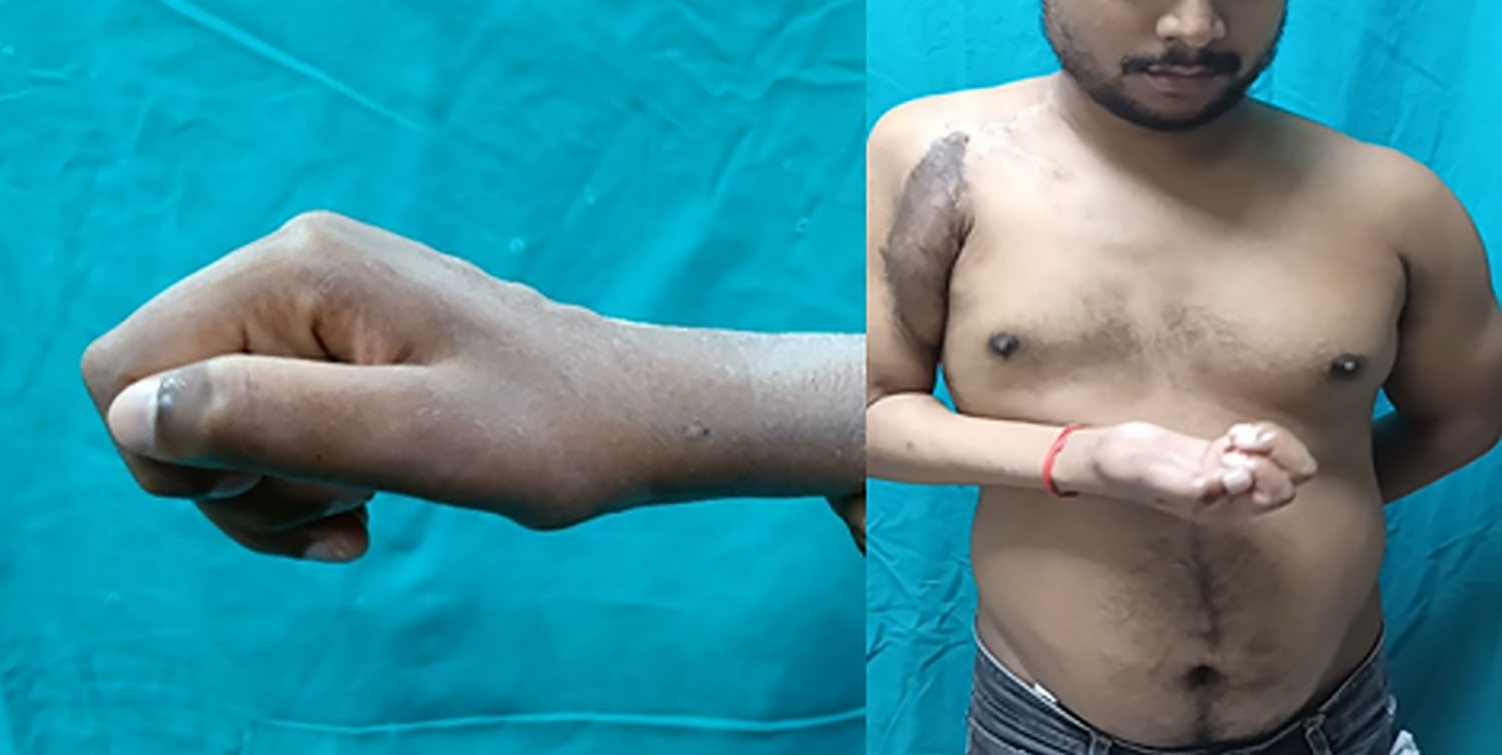
Two years after wrist arthrodesis in a patient with brachial plexus palsy, the transferred functioning free muscle at the wrist is more stable, and finger flexion is strengthened

Even though patients can function well with their dominant hand fused in the neutral position, 10–15 degrees of wrist extension and minimal ulnar deviation are often preferred [4]. Clayton suggested a neutral posture with an ulnar deviation of around 10° [28]. However, Larsson noted that a little extension and ulnar deviation boosted grip power [29]. On the other hand, the fusion posture did not influence grip strength or contentment, according to Hinds et al. [30]. Our study’s average postoperative hand position of patients following wrist arthrodesis was 17.5 degrees of extension and 6 degrees of ulnar deviation. However, in brachial plexus injury patients, we avoid extension of more than 15 degrees as it impairs the hand release function [25] (Fig. 6).

The subjective VAS incorporated questionnaire [15] used in the study population was also used by Terzis and Barmpitsioti to assess wrist functionality following fusion [25]. The questionnaire reported a good outcome for all patients in the follow-up in our study. Though the subjective questionnaire employed in this research has several drawbacks, it is still possible to utilise it to determine a patient’s perspective after surgery, as described in Table 2. In our study of patients who underwent wrist fusion, the Disabilities of the Arm, Shoulder, and Hand (DASH) questionnaire was not used because it evaluates the patient’s overall functional state and is not specific to wrist function.

The failure to perform a preoperative functional assessment and the retrospective nature are the limitations of our study. Further, we had no control group, such as those operated with an AO arthrodesis plate or using an iliac crest bone graft, and evaluated a heterogeneous sample group. Despite these limitations, our results of wrist fusion with a trapezoidal cortico-cancellous strut graft and a reconstruction plate have given us good results. It is a simple technique with no implant prominence risk and is advantageous over bone grafts from distant areas. Our study documented that a low-profile reconstruction plate is reliable and provides compression and stable fixation achieving radiological union without complications.
